# Recurrent Pericarditis: A Rare Adverse Effect of Mesalamine

**DOI:** 10.7759/cureus.33661

**Published:** 2023-01-11

**Authors:** Pratul Karki, Ariya Kunwar, Nitish Sharma, Megha Dogra

**Affiliations:** 1 Internal Medicine, MultiCare Good Samaritan Hospital, Seattle, USA; 2 Internal Medicine, Nepal Medical College, Gokarneshwar, NPL; 3 Cardiology, Saint Vincent Hospital, Worcester, USA; 4 Internal Medicine, Bassett Medical Center, Cooperstown, USA

**Keywords:** atypical chest pain, chest pain, inspiratory dyspnea, dyspnea, mesalamine, recurrent pericarditis

## Abstract

Inflammation of the myocardium (myocarditis), pericardium (pericarditis), or both (myopericarditis) is a rare but potentially lethal side effect of mesalamine, a drug widely used in the treatment of inflammatory bowel disease (IBD).

A 64-year-old female with a history of ulcerative colitis on mesalamine presented with dyspnea and chest pain. The patient was found to have mild to moderate pericardial effusion with signs of pericarditis. Further workup revealed positive rheumatoid factor, antineutrophil cytoplasmic autoantibody, cytoplasmic (c-ANCA), and antinuclear antibodies (ANA), raising suspicion for rheumatoid-associated pericarditis. She was discharged with a prednisone taper and an outpatient rheumatology follow-up. However, the patient presented again in three months with similar complaints and was found to have recurrent pericarditis. On this admission, mesalamine-induced pericarditis was considered in the differential diagnosis, and it was stopped on discharge. On a three-month follow-up, the patient showed complete resolution.

Mesalamine-induced pericarditis is a rare side effect of this drug, and prompt recognition and appropriate intervention are important to prevent the progression of the inflammation and avoid adverse cardiovascular outcomes. The association of IBD with extra-intestinal cardiac manifestations can delay early diagnosis and treatment.

## Introduction

Ulcerative colitis (UC) is an inflammatory bowel disease (IBD) that primarily involves the bowel and is associated with extra-intestinal manifestations in other organ systems. Cardiac involvement (pericarditis, myocarditis, and myopericarditis) is a rare and life-threatening manifestation if left untreated. Mesalamine (mesalazine) is a 5-aminosalicylic acid (ASA) compound that is the first-line treatment for patients with mild-to-moderate UC. Myopericarditis, a side effect of mesalamine, a drug widely used in the treatment of IBD, is an infrequent but potentially lethal complication [1]. Timely diagnosis and intervention are critical for preventing inflammation and avoiding long-term cardiovascular side effects [1].

## Case presentation

A 64-year-old female with UC on mesalamine (started one year ago) presented to the hospital with dyspnea and chest pain. The patient had been having recurrent episodes of chest pain over the last three weeks. The pain was located in the precordium without any radiation to the jaw or left arm. The episodes started spontaneously at rest and were exacerbated by exercise and deep breathing.

**Figure 1:**
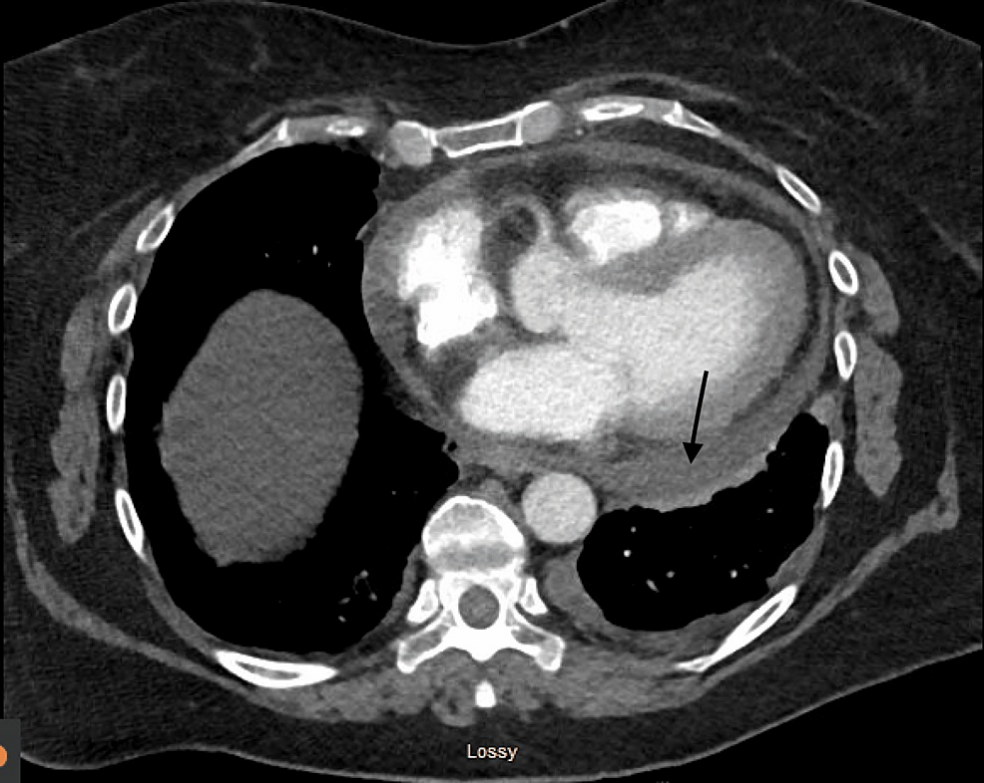
CTA chest with mild to moderate sized pericardial effusion CTA: computed tomography angiography

The patient’s pain was only relieved after rest and morphine. This was accompanied by progressively worsening shortness of breath with exertion. She also reported night sweats; however, she did not report any fever or recent upper respiratory tract infections. The patient had no history of coronary artery disease and no risk factors for the disease. She had an irregular, rapid rhythm on the exam, but no murmurs or friction rubs were found. No rashes were noted either. The labs on presentation were consistent with an elevated D dimer of 1740 ng/ml, an erythrocyte sedimentation rate (ESR) of 71 mm/hr, and a negative troponin. The electrocardiogram revealed atrial fibrillation, rapid ventricular response, and nonspecific ST and T wave abnormalities. An echocardiogram showed normal left ventricular size, systolic function, and trace pericardial effusion. A CT angiogram showed mild to moderate pericardial effusion along with irregular pericardial thickening and patchy areas of fat stranding, which was concerning for pericarditis, and a small left pleural effusion (Figure 1).

She was started on colchicine and naproxen. Autoimmune workup revealed positive antinuclear antibodies (ANA), antineutrophil cytoplasmic autoantibody, cytoplasmic (c-ANCA), and proteinase 3 (PR 3), after which rheumatology was consulted. The patient was discharged with a prednisone taper per rheumatology recommendations and an outpatient follow-up for possible rheumatoid arthritis (RA)-related pleuro-pericardial effusion.

The patient presented again after three months with continued exertional dyspnea and chest pain. Rheumatology reevaluated the patient and concluded that RA was less likely to be the cause of pericarditis given the absence of joint pain or swelling and no response to steroids. ANA was likely a false positive, and c-ANCA and PR-3 positivity were attributed to mesalamine use. A suspicion of mesalamine-induced recurrent pericarditis was raised. Gastroenterology was consulted, and they were in agreement to stop mesalamine.

The patient’s symptoms significantly improved after discontinuing mesalamine. She was evaluated in the clinic for three weeks, followed up at three months after discharge, and achieved complete recovery. She was switched to adalimumab for UC and did not have any further episodes of pleuro- pericarditis.

## Discussion

Mesalamine has long been the cornerstone of IBD treatment, especially for milder forms of UC [1]. Cardiotoxic manifestations in the form of myocarditis, pericarditis, or both are rare and life-threatening side effects of mesalamine use. Sulfasalazine is composed of mesalamine attached to sulfapyridine via an azo bond, which is cleaved by azoreductase from bacteria in the small bowel and colon. Sulfapyridine, thus released, is inactive; however, it is absorbed in the large intestine and is a major reason for hypersensitivity reactions and side effects associated with sulfasalazine.

The mechanism of 5-ASA-related cardiac side effects is not clearly known, and the proposed pathophysiology includes direct toxic effects and humoral or cell-mediated mechanisms [2]. Due to the lack of distinctive pathognomonic features of mesalamine-induced pericarditis, limited literature, and association of IBD with extra-intestinal cardiac manifestations, the diagnosis is challenging.

The diagnosis of mesalamine-induced myocarditis and pericarditis is made by ruling out other common causes of myocarditis and pericarditis and by observing the resolution of symptoms after cessation of treatment. Generally, the clinical features are seen within one to two weeks of starting mesalamine, and they can persist for up to four weeks or more [3,4]. Symptoms include fever, chest pain, dyspnea, fatigue, and generalized weakness. Tachycardia and pericardial rub might be examination findings in some patients [3,5]. Initial EKG findings are nonspecific ST segment changes or T-wave changes (flat, depressed, or elevated), with elevated T waves being more common [3].

Lab studies can show leukocytosis, elevated ESR/C-reactive protein (CRP), and elevated troponin [3,6]. Some patients can show elevated pro-brain natriuretic peptide (proBNP). Imaging studies, including an echocardiogram and a CT chest scan, can reveal left ventricular systolic dysfunction in addition to pericardial effusion with or without tamponade [5].

Multiple cases of mesalamine-induced myocarditis, pericarditis, and pleural and pericardial effusions have been reported in the literature. Recurrent attacks of myopericarditis have been reported with continued exposure to mesalamine. No dose-effect relationship has been demonstrated in the literature. For confirmation of the diagnosis of drug-induced inflammation, discontinuation of the 5-ASA product is generally enough [7]. A short course of corticosteroids has been tried for symptom management. It is unclear whether the steroid treatment reduces the time to resolution of symptoms because the time course is comparable with that in reports of discontinuation only [7]. Our patient was discharged with a prednisone taper without stopping mesalamine on the first admission, but required readmission for recurrent pericarditis despite being on prednisone. The pericarditis completely resolved after mesalamine was stopped.

IBD (UC and Crohn’s disease) is known to be associated with extra-intestinal manifestations, affecting multiple organ systems. These can affect the musculoskeletal system, eye, skin, hepatobiliary, hematopoietic, pulmonary, and cardiac systems. Cardiac manifestations include myocarditis and pericarditis. Extra-intestinal manifestations are found on initial presentation in less than 10% of patients, and 25% of patients develop extra-intestinal manifestations in their lifetime [8]. These manifestations can have a variable presentation during the course of the disease. Extra-intestinal manifestations tend to follow the clinical course of the colitis, with the exception of primary sclerosing cholangitis, uveitis, and ankylosing spondylitis. Pericarditis IBD is rare and poses a diagnostic challenge, especially with patients that are on the 5-ASA regimen [9].

## Conclusions

Mesalamine-induced pericarditis and myocarditis are rare but life-threatening complications. Early recognition is vital to prevent progression. There is no single lab test to prove mesalamine as the cause of pericarditis. It is a diagnosis of exclusion and other common causes of pericarditis should be ruled out first. The association of IBD with extra-intestinal cardiac manifestations of myocarditis and pericarditis poses a diagnostic challenge when the patients are on mesalamine. Physician awareness is necessary to differentiate between IBD-associated pericarditis and mesalamine-associated pericarditis for accurate diagnosis and management. Patients should be educated about the cardiovascular side effects of mesalamine and advised to seek medical attention if cardiac symptoms arise. 
